# A New Use for Telepathy

**Published:** 1893-01-21

**Authors:** 


### Contemporary Theory and Research.

### A NEW USE FOR TELEPATHY.

In the "Revue des Deux Mondes" M. Paulhan discusses some of the more practical aspects of what he terms the true hallucination. He argues that since all thought is accompanied by a movement of the brain, this movement may be transmitted to the surrounding ether, and communicate itself to other brains at a considerable distance from the point of departure, by reassuming its original form. The practical outcome of this process is the case of the country doctor who possessed a marvellous "subject" with a telepathic gift. When called out suddenly to a distance at night, the doctor would apply to his "subject," apparently with the best results, in order to ascertain the patient's disorder, and be able to take with him the necessary drugs. M. Paulhan is a firm believer in the future of telepathy, holding "that this old world of ours has no doubt still many surprises in reserve for us."

